# Abuse against elderly in India – The role of education

**DOI:** 10.1186/1471-2458-14-336

**Published:** 2014-04-09

**Authors:** Vegard Skirbekk, KS James

**Affiliations:** 1 Norwegian Institute of Public health, Pb 4404, Oslo N-0403, Norway; 2 International Institute for Applied Systems Analysis (IIASA), Schlossplatz 1, Laxenburg A-2361, Austria; 3 Institute for Social and Economic Change, Nagarabhavi, Bangalore 560072, India

**Keywords:** Elderly abuse, Violence, India, Education

## Abstract

**Background:**

Abuse against the elderly is recognized as an important challenge to elderly health, but its determinants are not yet well understood. We present findings from a new dataset which covers a representative sample of the population aged 60 years and above from seven Indian states across India – all of which have a higher proportion aged 60 plus compared to the national average. Earlier studies suggest that schooling levels can be relevant in determining the level of abuse against seniors. This study focuses on the role of education on the prevalence of elderly abuse in India.

**Methods:**

We conduct an analysis of cross sectional primary data that contains information on elderly abuse. The households in the sample were randomly selected from the seven demographically oldest states in India. These states are Himachal Pradesh, Kerala, Maharashtra, Odisha, Punjab, Tamil Nadu and West Bengal. A total of 9852 elderly from 8329 households were interviewed. The statistical analysis is based on logistic regression to understand the independent relation of education with abuse against the elderly.

**Results:**

Our findings reveal that 11% of 60+ year olds have experienced at least one type of elderly abuse (Physical 5.3%, Verbal 10.2%, Economic 5.4%, Disrespect 6%, Neglect 5.2%). The most common perpetrator is the son, who is reported to be responsible for the abuse among 41% of male victims and 43% of female victims. Formal education among elderly beyond a certain level (8 years) has a strong relation with reduced violence against elderly.

**Conclusions:**

Our findings suggest that level of schooling among elderly is strongly negatively related to abuse against them*.* More members in the household reduces the chance of abuse while having a greater number of children increases the chance of abuse (neglect and verbal abuse). We find that education even after controlling for wealth and other relevant variables is the factor that most consistently lowers elderly abuse. However, the relation of education to abuse is limited to those with more than 8 years of schooling. This suggests that the ongoing educational expansion beyond the basic schooling years in India may lead to a decline in the incidence of elderly abuse.

## Background

A lack of formal education has been shown to be related to a greater risk of elderly abuse [1]. This is particularly important in the Indian setting as education levels of older Indians are very low; it has been estimated that 73% of persons above 60 in India are illiterate [2]. In the present study we attempt to ascertain which facet of education is most strongly related to elderly abuse. There are a number of potential mitigating factors within the rubric of “education”. These include the ability to effectively respond to situations where abuse occurs. It could also include having certified skills (e.g., in the form of diplomas) demonstrating successfully completing a longer period of schooling, which may for instance increase the likelihood of being respected. Education levels may also increase one’s respect of seniors and decrease the likelihood that they will be subject to abuse. The results from this study could have important implications for policy aimed at reducing the prevalence of abuse. For instance, if cognitive functioning is found to play a more causal role, mental functioning could be augmented through programs meant to raise activity levels at older ages; if extended periods of schooling are found to be more causal, policies mandating a longer compulsory education could be more effective in reducing abuse.

Whether to invest in rising formal schooling levels is an important policy consideration. Unlike factors that are difficult to plan or influence by policies (such as changes in family structures, economic inequality and cultural changes), schooling is mostly determined by governmental choice. In India, schooling policies of the 20th century were more focused on creating a relatively small number of elite schooling institutions while universal, or even majority, school coverage was not achieved. This implies that large shares of the currently older population have little or zero formal schooling, while a minority has relatively long schooling. Also today, schooling levels are relatively low in India compared to other growing economies. Nevertheless, school levels can be an important determinant of elderly welfare decades later, including abuse against elderly.

Abuse against the elderly is recognized as a fundamental challenge to elderly health in India [3,4]. Nonetheless, a systematic prevalence survey covering states across the whole nation has so far not been produced. In the current study, we define being elderly as being above 60 years. We acknowledge that this represents only one way of defining being “elderly”, and that other cut-off ages could have been used [5]. We present findings from a new dataset [6] which covers a representative sample of the population aged 60 years and above from seven Indian states across India – all of which have a higher proportion aged 60 years and above compared to the national average. These states are Himachal Pradesh, Kerala, Maharashtra, Odisha, Punjab, Tamil Nadu and West Bengal.

Elderly abuse varies in form and severity. It includes physical, sexual and verbal maltreatment as well as financial exploitation and neglect [7]. Elderly abuse can have several health consequences including an elevated risk of depression, anxiety, self-neglect, physical inactivity and mortality [8,9].

Significant levels of elderly mistreatment have been identified in nations across Asia [10-13]. A study from Chennai (N = 400) found 14% prevalence of elderly mistreatment, where chronic verbal abuse was the most common followed by financial abuse, physical mistreatment, neglect and injuries from burns [14]. In another Indian study (N = 1000), 4% of the elderly respondents reported physical abuse [15].

In India, most elderly do not have health insurance and do not receive pensions – about 90% of the Indian elderly lack a formal pension scheme or social security arrangements [2]. Further, one study found that in 2001, 55% of women over 60 years are widows – which makes this demographic group particularly vulnerable since having a male guardian can be important for their safety and status within the Indian social context [16]. Due to poor social security coverage, the elderly tend to depend upon their children for their daily needs and care during old age. As such, elderly living in the household could represent a significant burden in terms of finances and time-use for the families who support them [3,17].

Family members have been found to be the most common perpetrators of elderly abuse [4]. India is traditionally a patriarchal society, and sons are expected to take care of the elders within the family. Seventy-five percent of the elderly live with sons and only 3 percent live with daughters [18]. Given the fact that the elderly tend to live with sons, abuse that takes place within the family is more likely to be carried out by sons and daughter-in-laws.

Provision of care to senior dependents can cause high levels of mental and physical stress, and may for instance raise the risk of depression [19]. Care provision has also been found to augment the risk of suffering verbal and physical abuse as well as shame and sleep deprivation, particularly for those who take care of the cognitively and physically impaired [20]. Furthermore, care provision can be associated with heavy financial demands and time requirements from the care-provider [21].

## Methods

We use a 2011 national level survey conducted across seven states in India by the United Nations Population Fund (UNFPA): “Building Knowledge Base on Population Ageing in India”. The survey gathered information from seven states in India and consisted of elderly households. Being “elderly” in this survey was defined as those aged 60 and above, consistent with the definition of the Government of India in the provision of various services. In total, 8792 households were selected for this survey. Out of these, 8329 (94.7 percent of the households) of the interviews were completed. There were 10604 elderly in the 8329 households. Of these, 9852 elderly were interviewed (a response rate of 92 percent). The states surveyed are Kerala and Tamil Nadu from the south, Maharashtra from the West, Orissa and West Bengal from the east and Punjab and Himachal Pradesh from the north. While all surveyed states are demographically older than the national average, within this sample there are significant variations in per-capita income and demographic development. For example, Punjab and Maharashtra are economically relatively advanced, while Orissa is one of the poorest states in the country. Kerala has the highest proportion of elderly but also well-developed demographic indicators, including relatively low fertility. Other states fall in between both in terms of economic and demographic indicators.

We conducted a survey asking elderly household members several questions to understand their situation and potential level of abuse. The survey first determined whether the elderly faced any type of abuse after the age of 60. If they have experienced any violence, it included a questions on whether the abuse was recent (taking place during the preceding month). For those who had experienced abuse, the survey asked more specific questions regarding five types of maltreatment including physical, verbal, economic, disrespect and neglect as well as the source of such violence. Finally, any type of physical or emotional abuse experienced by the elderly in the preceding month is also noted. The working definition of these types of abuse is based on the WHO definition. These details were provided to the investigators in a manual which explained the questionnaire and provided specific survey-related training. We analyzed the information derived from the surveys to build a comprehensive understanding of elderly abuse in India.

The survey took particular care to facilitate a high quality of responses. All investigators were instructed to ensure that questions regarding abuse were not asked when other family members were present. Based on a review by the Internal Ethics Committee at the Institute for Social and Economic Change, the survey employed in this experiment was not found to collect any biomarkers or other sensitive information that could be used to the detriment of the participants. In consideration of the information being collected through the questionnaire, the Institute for Social and Economic Change waived the need for ethics approval for the study. We ensured the anonymity of all participants and all data gathered was kept strictly confidential, consistent with the stipulations as enumerated in the Helsinki Declaration. Informed consent was obtained from all individuals prior to participation in the experiment. If the elderly respondent was unable to respond due to incapacitation or ill health, the interview was conducted through a proxy respondent. In the case of proxy interviews (where family members were asked about the situation of the respondent - this was the case for 73 cases), information on abuse was not gathered. Following these limitations, the number of respondents who answered questions on abuse was 9779 individuals.

Since one of the objectives of the study is to understand the relationship between educational level and prevalence of abuse, we classified the educational level of elderly into four groups. The overall educational level among elderly is very poor. The highest grade has been classified as eight years of schooling and above. In our sample, 13 percent of women and 36 percent of men have eight or more years of schooling. 35 percent of the men and 66 percent of the women are illiterate. Literacy level is determined based on whether an elderly with less than 6 years of schooling is able to read out words printed in a card.

The study has shortcomings, including that abuse could be underreported due to cultural reasons. Further, certain indicators, like children’s characteristics, were not included in the model. The children’s education was difficult to assess. When there are several children, one would need to decide whose education level should be considered in the analysis (as we do not know which child the perpetrator of abuse was in the case of multiple sons or daughters). Second, when no children were staying with the elderly, information on children was not collected and therefore we do not have this information in many cases.

The statistical analysis is based on logistic regression to understand the independent association of education with abuse against the elderly. Several other factors that are likely to be associated with abuse are also included in the model such as marital status, caste, formal education, cognitive function, wealth, employment status, place of residence, household size, living children and property/asset ownership. The classification of caste is based on Scheduled Caste and Scheduled Tribe (SC/ST), Other Backward Classes and Other Caste Groups. The SC/ST are socially and economically among the most least developed groups in India followed by OBC (Other Backward Classes). The caste group denoted as “others” refer to those at the higher end of the caste ladder. As the reliability of income data obtained from surveys are of suspect in India, we have computed an asset ownership index by combining several assets and amenities within the household.

## Results

About 11 percent of the elderly have experienced some form of abuse after turning age 60, see Table 1. Women experience a higher prevalence of most forms of abuse than men, except for physical violence. Around 4 percent have experienced physical abuse. Among other forms of abuse, verbal abuse appears to be the most serious problem with around 10 percent of the elderly experiencing it. It is possible that verbal abuse could be a major pathway by which forceful transactions of property, money, etc. are achieved within the family. The experience of other forms of abuse like economic, disrespect and neglect amounts to around 5 percent. Furthermore, the majority of those who commit the abuse come from within the family.

**Table 1:** Percentage experiencing different form of abuse after turning 60 years of age, India, 2011

**Type of abuse**	**Men**	**Women**	**Percent of victims abused by family members**
Physical	4.6	4.0	66.7
Verbal	9.7	10.7	68.0
Economic	5.1	5.7	68.3
Disrespect	5.3	6.6	55.9
Neglect	4.2	6.0	65.2
Ever experience of abuse	11.0	11.7	NA
Current experience of abuse	5.2	6.2	
No. of elderly	4,635	5,144	

Note: NA = Not applicable.

Our findings suggest that nearly 6 percent of the respondents are currently experiencing abuse (in the one month preceding the survey), where women are one percent more likely than men to experience abuse. The current experience of physical violence is around 3 percent. While there is around a 5 percentage difference between past and current experience of any abuse, the differences are narrower in the case of physical violence with only a single percentage difference. This indicates that those subjected to physical violence experience it on a more regular basis.

Education seems to have a protective relationship to violence perpetuated toward the elderly. In India, the average educational level among the elderly is very low, where more than half of the respondents did not attend schooling. However, this study found that just having a few years of education was associated with lower abuse prevalence (Table 2). The experience of abuse is systematically reduced with increases in educational level. The lowest abuse levels are found for those with at least 8 years of education. Thus, education seems to be a protective factor decreasing levels of abuse experienced by older individuals.

**Table 2:** Percentage of elderly experiencing abuse after turning 60 years of age by type of abuse and level of educational, India, 2011

**Experience of abuse**	**Years of education of elderly**					Chi square test*
	**Illiterate**	**1-4 years**	**5-7 years**	**8+ years**	**Total**	
Physical	5.3	5.6	3.0	1.9	4.3	63.8 (0.000)
Verbal	12.6	11.2	9.2	4.8	10.2	115.7 (0.000)
Economic	7.1	5.8	4.3	2.0	5.4	89.4 (0.000)
Disrespect	7.5	7.3	4.8	2.5	6.0	77.9 (0.000)
Neglect	6.9	6.0	3.5	1.7	5.2	91.9 (0.000)
No. of elderly	4,501	1,252	1,313	2,660	9,726	

Note: *the figures in the bracket are the p values.

We also considered several other potential determinants of abuse against elderly. This includes a measure of cognitive ability (immediate recall) as a possible indicator which could be related to abuse. Unlike formal education, cognitive ability was not found to be very important for current abuse but has a significant relationship with the experience of past abuse.

Table 3 presents the percentage experiencing abuse by levels of schooling and other major socio-economic characteristics. Caste shows an interesting pattern as both the lowest castes (*Scheduled Castes* and *Scheduled tribes*) as well as the *Others* have a high incidence of abuse, while the caste that comes in middle in terms of socio-economic standing, *Other Backward Classes*, has the lowest incidence of abuse. This is true in all educational categories. Education appears to have a significant impact on reducing violence irrespective of caste groups.

**Table 3:** Percent of elderly experiencing abuse by levels of schooling and selected background characteristics, India, 2011

**Characteristics of elderly**	**Levels of schooling among elderly**			**Total**
	**Illiterate**	**1-7**	**8+**	
**Caste**				
SC	13.4	13.2	3.5	12.1
ST	13.4	17.6	10.2	13.9
OBC	9.8	8.6	3.3	8.0
Others	17.9	14.6	7.2	13.9
**Ownership of land**				
No	14.5	12.0	4.7	11.9
Yes	12.1	11.6	6.6	10.6
**Wealth index**				
Lowest + second (40%)	15.5	16.7	9.8	15.3
Middle (20%)	10.8	8.8	4.6	8.7
Fourth + highest (40%)	11.4	7.5	4.6	7.6
**Cognitive ability (Immediate recall of words)**				
0-5	14.0	12.7	4.2	11.9
6-10	11.8	10.7	7.4	9.5
Total	13.8	11.8	5.5	11.4

Several studies have indicated that property ownership can affect the likelihood of being a victim of abuse [22,23]. The motivation for such abuse, particularly within the family, would be to ensure property transfer to the members perpetuating abuse. Throughout India, different forms of arrangements exist for the transfer of property from elderly to children. Although the inheritance law generally mandates equal distribution of property (land and house) among both sons and daughters, in practice, sons inherit the major part. The data suggest that the share of elderly experiencing abuse is similar among those with and without landholding. However, the level of education appears to make significant difference, both among elderly with and without landholdings.

We have also included a wealth index using asset holdings as well as amenities within the household. The wealth index has a close connection with property holding. The methodology used to compute wealth index is similar to the one developed by Demographic Health Surveys [24]. The sample was divided into five quintiles, where the lowest represents the poorest wealth quintile. But it was further collapsed into three groups here for ensuring adequate sample size in each cell. The experience of abuse is significantly higher among poor sections of the population and as wealth increases, the incidence of abuse is significantly mitigated. While around 15 percent of the elderly in the lowest 40 percent wealth quintiles experienced abuse, it is only 8 percent in the case of highest 40 percent wealth quintile group. Education appears to reduce the experience of violence in all the wealth quintile groups but the magnitude is slightly less among lower wealth quintiles.

The survey also measured the cognitive ability of the elderly in terms of immediate recall of 10 words. The experience of violence among elderly with lower cognitive ability is around 2 percent higher. The exception was only for the highest educational category where the experience of violence is marginally higher among those with higher cognitive abilities.

## Discussion

We conducted an analysis to understand the relationship of education with other types of abuse after controlling for other relevant characteristics, using a logistic model. The dependent variable is experience of any type of abuse after turning 60 years old. The analyses were done for every type of abuse but only the results for experience of any types of abuse is presented (see Table 4).

**Table 4:** Results of logistic regression analysis of elderly who experience any abuse or violence after turning age 60 years, India, 2011

**Characteristics of elderly/elderly households**	**Odds ratio**	**SE**	**P-value**
**Constant**	0.197*	0.265	0.000
**Age group (Ref: 60–69)**			
70+	1.137	0.082	0.117
**Sex (Ref: Male)**			
Female	1.007	0.112	0.952
**Marital status (Ref: Currently married)**			
Widowed	1.281*	0.093	0.008
Others	3.135*	0.248	0.000
**Caste/tribe (Ref: Forward caste)**			
SC/ST	0.787*	0.094	0.011
Other backward caste	0.739*	0.098	0.002
**Education (Ref: Illiterate)**			
1–4 yrs	1.031	0.112	0.786
5–7 yrs	0.863	0.120	0.219
8+ yrs	0.480*	0.126	0.000
**Employment (Ref: Never worked)**			
Worked earlier	1.298	0.115	0.023
Currently working	1.634*	0.126	0.000
**Wealth index square**	1.064	0.036	0.088
**Ownership of land (Ref: No land)**			
Yes	0.709*	0.090	0.000
**Place of residence (Ref: Rural)**			
Urban	0.692*	0.083	0.000
**No. of members in HH (Ref: One member)**			
2 member	0.616*	0.180	0.007
3+ member	0.431*	0.160	0.000
**No. of children alive (Ref Less than or equal to 2)**			
3–4	1.413*	0.090	0.000
5+	1.584*	0.102	0.000
**Cognitive score (Ref: Less than or equal to two words)**			
Medium (3/5 words)	0.985	0.108	0.886
High (6/10 words)	0.998	0.138	0.988
**States (Ref: Himachal Pradesh)**			
Punjab	0.709*	0.090	0.000
West Bengal	0.933	0.137	0.615
Odisha	0.655*	0.150	0.005
Maharashtra	0.743	0.149	0.045
Kerala	4.126*	0.121	0.000
Tamil Nadu	0.348*	0.194	0.000
Log Likelihood square	5167.61		

Note: *Significant at less than the 0.01 level.

The result shows that with respect to schooling, only those having at least eight years or more education have a significantly lower likelihood of experiencing of abuse. Hence, it is not by merely having some education but by possessing a critical minimum level that elderly are able to reduce or avoid abuse. Education has a significant negative relation to all types of abuse. This implies that the inevitable rise in education, following the greater educational attainment of later born cohorts, could lead to a decrease in the prevalence of elderly abuse in India. Results of the regression analysis are consistent with those indicated by the bi-variate relations.

There are no significant age or sex differences in violence against the elderly. However, those currently married have less experience of abuse relative to widows and others (including never married, separated and divorced). This clearly shows that elderly who are alone or without spousal support are more vulnerable to abuse. Unlike in the case of bi-variate analysis, the regression result shows experience of violence higher among forward caste (other caste group) compared to lower caste after controlling for other factors. This is surprising given the fact that there are close linkages between caste and class and therefore the result shows indications of high instance of violence among richer sections.

The square of wealth index score is used to measure economic status of the household. But it did not show a significant relationship with elderly abuse. Wealth index, however, measures only the household economic status and not that of elderly. The land holding among elderly, however, indicates lower chances of abuse. Possessing either a given level of education or economic capital in terms of property has the effect of reducing violence against elderly in India. Less violence experienced by urban residents compared to their rural counterparts may also be partly due to the higher social and economic development in their regions. Similar findings can also be observed considering the relationship between work status of elderly and experience of violence. Work among the elderly in India is often associated with poverty and as such those who are working also experience a higher incidence of violence.

Two other important demographic variables considered for the analysis are household size and number of children. We found that both these indicators are important but provide conflicting results. While the number of household members reduces the prevalence of abuse, we found the opposite impact on the number of children. As the number of children in the household increases, so does the risk of abuse. Perhaps different mechanisms are operating for these indicators to lead in opposite directions. One potential explanation could be that a higher number of household members will reduce abuse as peer pressure precludes individuals of abusive behavior when many are present. On the other hand, when there are more children, the possibility of disputes related to for instance sharing property can increase and could subsequently lead to greater abuse against the elderly (even when children are not residing within the household). Moreover, the number of children born for the elderly has a direct linkage with the educational level among them. Therefore, this implies that the ongoing rapid demographic changes along with educational improvement have the potential of reducing the incidence of abuse among elderly in the future.

Higher cognitive abilities do not have a significant impact on the risk of being subject to abuse, in spite of the fact that cognitively more fit individuals are generally healthier, earn more and live more social lives [5]. It may be because cognitive abilities could have opposing effects. On the one hand, cognitively adept individuals are better able to understand, remember and express cases of abuse – thereby reporting a greater number of the cases of elderly abuse, while higher cognition can also be related to avoiding abuse – and the net relationship could be insignificant.

Most abuse against the elderly takes place within the home. Table 5 presents information on individuals within and outside the family who have inflicted abuse on the elderly. We observed that sons and daughters-in-law are the offenders in over 60 percent of elderly abuse cases in India. Around a quarter of abuse instances are inflicted by others, including servants and outsiders. Daughters and grandchildren contribute to only a small proportion of total abuse.

**Table 5:** Percentage of elderly experiencing abuse by person abusing and marital status of the elderly, India, 2011

**Person abusing**	**Men**			**Women**		
	**Currently married**	**Others**	**Total**	**Currently married**	**Others**	**Total**
Spouse	3.5	0.0	3.0	3.7	0.0	1.2
Son	42.0	34.4	40.9	47.2	41.1	43.0
Daughter	1.2	0.0	1.0	2.2	5.0	4.1
Son-in-law	0.0	0.0	0.0	1.2	3.2	2.6
Daughter-in-law	13.7	23.9	15.2	23.8	16.9	19.0
Grand children	1.0	0.8	1.0	2.4	5.0	4.2
Other	38.6	41.0	38.9	19.6	28.9	26.0
No. of elderly	175	34	209	86	198	284

## Conclusions

Respect for those old and frail and protection of the weak are two ideals that many societies often pride themselves on. However, evidence suggest that abusive treatment of elderly is relatively common across different societies and cultures [16,25,26]. Being subject to abuse by caregivers can be a humiliating and despairing situation for the victims. Elderly abuse violates social norms of respecting elders and maintaining the family – and is therefore a topic that is subject to many taboos.

Inevitable population ageing implies that it is important to find ways to increase the well-being of the older population. In 2010, more than 7.6% of its population was aged 60 and above, and population projections suggest that India will have the world’s largest population of 60 plus by 2075, according to the UN medium fertility projection scenario [27]. India is estimated to already be older than several European countries if certain functional measures of health (related to cognitive functioning and education), as opposed to age, are used [5].

We find that 11% of 60+ year olds are subject to elderly abuse, which would equal a number of about 10 million victims in India (in 2010) in this age group for India as a whole, assuming that the findings from the seven states are nationally representative. The actual number of victims is likely to be greater as underreporting could be substantial since many elderly do not report abuse due to the stigma, feelings of shame, obligation to protect family name, fear of vengeful acts from perpetrators, or since many seniors have cognitive and physical impairments and may not be able to express any experiences of abuse [28-31]. Greater education may potentially affect the level of openness on abuse among the elderly. For instance, a greater educational level may increase one’s independence and one’s willingness to share information on such sensitive topics, including abuse. On the other hand, more education may also potentially be related to a greater importance of maintaining a family facade – and could potentially be related to a decreased willingness to share such information.

However, several aspects of ongoing societal change are associated with lower abuse level. A key finding is that even after controlling for wealth and other important factors, education is a factor that consistently lowers elderly abuse. However, the impact of education is limited to those with more than 8 years of schooling. This suggests that the ongoing educational expansion in India may lead to a decline in the incidence of elderly abuse.

While there is less certainty to which economic development and economic growth will spread to all members of society, there is less uncertainty about educational levels. There will be an inevitable rise in school levels along cohort lines. However, future developments depend greatly on schooling policies and choices. We did not find that having greater cognitive performance was associated with a lower incidence of elderly abuse. Our most striking finding regards the relation between formal education and elderly abuse. We find that education is the factor that most consistently lowers elderly abuse and those with more than 8 years of education are less likely to experience abuse compared to those with no education. This suggests that the ongoing educational expansion in India may lead to a decline in the incidence of elderly abuse. Education can improve one’s economic opportunities and give oneself greater status as well as increase one’s ability to take action to reduce potential abuse. In effect, policies that prolong education could be important in terms of reducing elderly abuse in India.

## Competing interests

The authors declare that they have no competing interests.

## Authors’ contributions

VS conceptualized the study and carried out part of the literature review and the interpretation. KSJ co-lead collection of the data and worked on interpretation of the results. Both authors read and approved the final manuscript.

## Pre-publication history

The pre-publication history for this paper can be accessed here:

http://www.biomedcentral.com/1471-2458/14/336/prepub
